# Management of acute acquired comitant esotropia in children

**DOI:** 10.22336/rjo.2023.16

**Published:** 2023

**Authors:** Mihaela Sorina Dragomir, Mircea Merticariu, Corina Ioana Merticariu

**Affiliations:** * Department of Ophthalmology, “Dr. Victor Gomoiu” Children’s Hospital, Bucharest, Romania; ** Department of Urology, CF2 Hospital, Bucharest, Romania; *** “Titu Maiorescu” University of Medicine, Bucharest, Romania

**Keywords:** acute acquired comitant esotropia, diplopia, intracranial disease, medial rectus recession, lateral rectus resection, strabismus surgery

## Abstract

**Aim:** This report aims to discuss and review the diagnosis and management of acute acquired comitant esotropia (AACE) in children and to add several cases to the limited literature available on this unusual condition in the pediatric population.

**Materials and methods:** We present two cases of AACE with large-angle deviations that were investigated and followed-up according to current recommendations. Both cases required strabismus surgery for AACE, but different procedures were chosen, with good postoperative results.

**Results:** Unilateral recession of the medial rectus and resection of the lateral rectus (R&R) were performed in one case and bilateral medial rectus (MR) recession in the other, with resolution of the diplopia and full recovery of binocular vision.

**Discussion:** Although isolated AACE is usually benign, studies have reported the presence of intracranial disease in up to 10% of cases, making it a potential first sign of an underlying serious pathology. Therefore, AACE should be investigated as a medical emergency and neuroimaging should be performed in all patients with unclear onset of AACE, as well as in those with associated neurological symptoms, such as headache, cerebellar imbalance, weakness, or nystagmus.

**Conclusion:** Acute acquired comitant esotropia (AACE) is an infrequent type of esotropia that usually appears in older children. It is characterized by esotropia and diplopia with acute onset. Neurological examinations and neuroimaging should be performed to exclude any potential intracranial disease. Treatment of AACE without underlying neurological disease is focused on managing the diplopia and resolving the esotropia. Strabismus surgery has good motor and sensory results and can successfully restore good binocular function.

**Abbreviations:** AACE = Acute acquired comitant esotropia, LR = lateral rectus, MR = medial rectus, PD = prism diopters, R&R = recession and resection, BSV = binocular single vision, PAT = prism adaptation test

## Introduction

Acute acquired comitant esotropia (AACE) is a specific form of strabismus that generally occurs in older children or adults and is distinguished by the sudden onset of a large angle non-accommodative esodeviation [**1**]. It is characterized by diplopia and esotropia that appear unexpectedly, in otherwise normal eyes, with normal eyeball movements, equal squint deviation in all directions of gaze and maintenance of a certain degree of binocular vision [**2**-**5**]. Esotropia can be intermittent or constant. In 1958, AACE was initially divided into 3 different subtypes, by Burian et al., on a small cohort [**2**,**6**-**9**]: Type I, known as the Swan type, which occurs after interruption of fusion, monocular occlusion, or loss of vision in one eye [**10**,**11**], Type II AACE, known as the Burian-Franceschetti type, which is characterized by low hyperopia, a minimal accommodative element and is precipitated by physical or psychological stress [**12**], and Type III, the Bielschowsky type, which is associated with myopia greater than -5.00 D, convergence spasm and divergence paralysis [**13**,**14**]. In a retrospective study based on 48 children, that took place from 2000 to 2013, Buck et al. reclassified AACE into 7 types: 

• Type I AACE, the Swann-occlusion related type, 

• Type II Burian-Francescheti, idiopathic, nonaccommodative, precipitated by physical or psychological stress, 

• Type III, acute accommodative AACE, characterized by hyperopia higher than 3.00 D and normal fusion; correction of the refractive error alone can adequately control this type, 

• Type IV, decompensated AACE, monofixation syndrome or esophoria, 

• Type V, less frequent, associated with intracranial pathology, most frequently a lesion of the posterior fossa [**7**,**14**-**16**], and the least common types of AACE, 

• Type VI, cyclic AACE,

• Type VII, secondary AACE. 

The most frequent types of AACE are Types III and IV, followed by Type II AACE. Type V was present in 6% of cases in the previously mentioned study, making it an important etiology to rule out [**15**-**18**].

Chunyan Cai et al. suggested in their study published in 2019 that there might be an anatomical particularity behind AACE. They conducted a study on 45 AACE patients, from 2011 to 2017 and found that the distance measured from the medial rectus insertion to the corneal limbus was shorter in patients with AACE [**1**,**19**,**20**]. 

There is no consensus regarding the etiology of AACE. However, Neena R et al. followed 15 cases for 6 months, during the COVID-19 pandemic, and observed that AACE can be triggered by excessive use of computers, tablets and smartphones, due to prolonged near point demands [**21**-**23**].

## Materials and methods 

We present two cases that were referred to our clinic with acute onset comitant esotropia. Comprehensive medical histories were taken, and we recorded onset, precipitating factors and associated symptoms. We assessed the deviation using prismatic cover/ uncover and alternate cover tests in all cardinal gaze positions. We performed a motility exam on all patients, to detect paresis, nystagmus or alphabetical syndromes. Visual acuity was measured in all patients, fusion potential and prism adaptation tests (PAT) were performed to fully assess the diplopia.

We measured cycloplegic refraction 40 minutes after administering 1% cyclopentolate eye drops. Two eye drops were administered at a 10-minute interval. Neurological examinations were performed in both cases, patients had brain and orbital magnetic resonance imaging and were monitored for at least 6 months after initial presentation. 

## Results

**Fig. 1:**
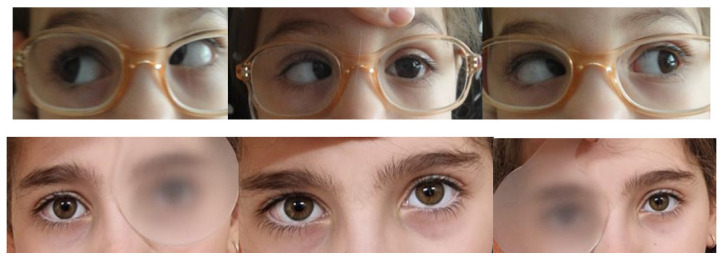
Case 1 - Motility exam before and after surgery. From the archive of the Department of Ophthalmology, “V. Gomoiu” Hospital, Bucharest, Romania

The first patient was an eight-year-old girl referred to our clinic by the neurologist for acute strabismus and diplopia that appeared during a period of emotional stress. By the time we examined her, 6 months after her symptoms appeared, she had undergone several eye exams that recommended eye patching and exercises in the synoptophore. Her visual acuity was 20/20 in each eye and her MRI was normal at the time of examination. She had a 50 PD esotropia with a small hypertropia in the right eye and was able to maintain fusion with 45-55 PD. Cycloplegic refraction showed a small hyperopia of +0.75 in the right eye and +1 in the left eye. We classified it as a type II AACE. A recession/ resection procedure was performed in the right eye, with 6.5 mm medial rectus recession and 8.5 lateral rectus resection. Her diplopia disappeared the first day after surgery. One month after surgery she was orthotropic, she regained binocular single vison for near and distance and stereopsis was normal (**Fig. 1**). Results were stable 6 months after surgery.

**Fig. 2:**
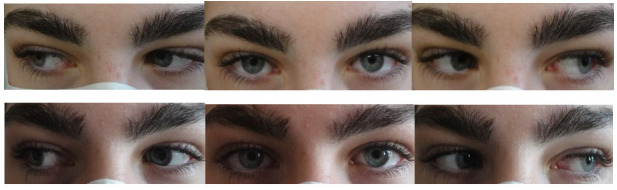
Case 2 - Motility exam before and after surgery. From the archive of the Department of Ophthalmology, “V. Gomoiu” Hospital, Bucharest, Romania

The second patient was a fourteen-year-old boy who was urgently referred to our clinic by the neurologists for sudden onset diplopia. The patient had a compensated hypothyroidism and described an episode of high fever one month prior to the diplopia. He had a small uncorrected astigmatism of -0.5 cylinder in both eyes and a Snellen corrected visual acuity of 20/20 in both eyes. The cover test showed 30 PD esodeviation for distance and near. Ocular motility was normal and prism adaptation test revealed good potential for fusion. His fundus examination revealed an elevated optic nerve head with blurry margins. Brain MRI was normal and ocular ultrasound showed drusen of the optic nerve head. We classified it as a type IV AACE, decompensated esophoria. He was prescribed 25 BO Fresnel prism to control the diplopia and was monitored for 6 months. Afterwards, a bilateral 5 mm medial rectus recession was performed. The first day after surgery he had 2 PD residual esotropia and no diplopia. One month after surgery he was orthotropic, had regained binocular single vision and normal stereopsis (**Fig. 2**). He was followed-up for one year with stable motor and sensory results.

## Discussion

AACE should be investigated as a multidisciplinary medical emergency by the ophthalmologist and the neurologist. MRI should be performed to rule out intracranial disease, however, there is still controversy regarding the best timing for neuroimaging, since there is no single sign or symptom that can predict the presence of underlying intracranial pathology [**15**]. Although isolated AACE is usually benign, studies have reported the presence of intracranial afflictions in up to 10% of cases, making AACE a potential first sign of an underlying neurological disease [**16**-**19**]. Upon extensive investigation, neurological involvement was ruled out in both our patients, although initially there was a high suspicion of underlying disease in at least one of them. We recommend neuroimaging in all patients with unclear onset of AACE or with associated neurological symptoms, such as headache, cerebellar imbalance, weakness, or nystagmus. 

The treatment was surgical in both our cases, with postoperative restoration of binocular function and resolution of the diplopia [**3**-**8**]. Two different surgical procedures were chosen, bilateral medial rectus (MR) recession in one case, and unilateral medial rectus recession combined with lateral rectus resection (R&R) in the other, with no proof of superiority of one surgical procedure over the other [**14**,**24**-**28**].

## Conclusion

Acute acquired comitant esotropia (AACE) is a specific type of strabismus that generally occurs after binocular vision has developed. Although most patients have no intracranial underlying disease, the possibility of a serious neurological affliction should be considered in all patients with AACE. 

The initial treatment in AACE is conservative and involves either prescription of glasses, prisms, eye patching or botulinum toxin injections. Surgery should be taken into consideration 6 months after the onset of the esotropia, if the deviation is stable. Strabismus surgery has good results from both motor and sensory points of view and can successfully restore binocular vision. 

**Conflict of Interest statement**

The authors state no conflict of interest.

**Informed Consent and Human and Animal Rights statement**

Informed consent has been obtained from the legal representatives of the individuals included in this study.

**Authorization for the use of human subjects**

Ethical approval: The research related to human use complies with all the relevant national regulations, institutional policies, is in accordance with the tenets of the Helsinki Declaration, and has been approved by the review board of “Dr. Victor Gomoiu” Children’s Hospital, Bucharest, Romania.

**Acknowledgements**

None.

**Sources of Funding**

None.

**Disclosures**

None.
